# Commentary: Abdominal Ultrasound and Its Diagnostic Accuracy in Diagnosing Acute Appendicitis: A Meta-Analysis

**DOI:** 10.3389/fsurg.2021.753405

**Published:** 2021-10-20

**Authors:** Jiangfeng Wu, Anli Zhao, Yun Jin

**Affiliations:** Dongyang Hospital of Wenzhou Medical University, Dongyang, China

**Keywords:** appendicitis, meta-analysis, ultrasound, abdomen, diagnosis

We read with great interest the manuscript of Fu et al. entitled “abdominal ultrasound and its diagnostic accuracy in diagnosing acute appendicitis: a meta-analysis” (1). The authors highlight the significant accuracy of diagnosis of abdominal ultrasound in patients with suspected acute appendicitis. We strongly agree with the authors about the importance of the abdominal ultrasound, but we would like to pay attention to several important missing aspects in the article.

First, in this meta-analysis (1), the author depicted that only studies adopting histopathology reports as the reference standard were included. But the reference standard of the included study was histopathology or 3 months of medical record follow-up if surgery was not performed (2), which was not consistent with what the author depicted. In the study by Tyler et al. (3), patients were classified as having appendicitis based on pathologic diagnosis, if available. If no pathologic diagnosis was available, a final CT read was used to classify the patient. So it might be not appropriate to include the two studies above in this meta-analysis.

Second, in the study by Khan et al. (4), a total of 223 pediatric appendectomies were performed, and the histopathology of eight was normal; 192 of 215 cases of appendicitis confirmed by histopathology were diagnosed by ultrasound, so the sensitivity of abdominal ultrasound in evaluating appendicitis by ultrasound in the study was 89%, which was different from the 86% reported by Fu et al.

Finally, in this meta-analysis, the overall sensitivity, specificity, positive likelihood ratio, and negative likelihood ratio were estimated at 77%, 60%, 2.62, and 0.45, respectively, demonstrating that abdominal ultrasound has a high false-positive rate (40%) and slightly high false-negative rate (23%) and should not be used for exclusion or inclusion of appendicitis, as the false-negative cases may progress to perforated appendicitis and peritonitis and result in a critical condition. But, in conclusion, the author demonstrated that abdominal ultrasound shows significant accuracy of diagnosis in patients with suspected acute appendicitis and is an effective diagnostic alternative to reduce the rate of unnecessary surgeries in acute appendicitis. We consider that the conclusion might be not appropriate. According to the overall results, patients suspected of appendicitis should be referred to more sensitive and specific diagnostic procedures, such as CT or MRI.

## Author Contributions

JW: concept and designed the study. AZ: drafting of the manuscript. YJ: proofreading, final editing, and guarantor of the manuscript. All authors read and approved the final version of the manuscript.

## Conflict of Interest

The authors declare that the research was conducted in the absence of any commercial or financial relationships that could be construed as a potential conflict of interest.

## Publisher's Note

All claims expressed in this article are solely those of the authors and do not necessarily represent those of their affiliated organizations, or those of the publisher, the editors and the reviewers. Any product that may be evaluated in this article, or claim that may be made by its manufacturer, is not guaranteed or endorsed by the publisher.

## References

[1] Fu J Zhou X Chen L Lu S. Abdominal ultrasound and its diagnostic accuracy in diagnosing acute appendicitis: a meta-analysis. Front Surg. (2021) 8:707160. 10.3389/fsurg.2021.707160 34262936 PMC8273278

[2] Crocker C Akl M Abdolell M Kamali M Costa AF. Ultrasound and CT in the diagnosis of appendicitis: accuracy with consideration of indeterminate examinations according to STARD guidelines. Am J Roentgenol. (2020) 215:639–44. 10.2214/AJR.19.22370 32406773

[3] Tyler PD Carey J Stashko E Levenson RB Shapiro NI Rosen CL. The potential role of ultrasound in the work-up of appendicitis in the emergency department. J Emerg Med. (2019) 56:191–6. 10.1016/j.jemermed.2018.10.034 30594351

[4] Khan U Kitar M Krichen I Maazoun K Ali Althobaiti R Khalif M. To determine validity of ultrasound in predicting acute appendicitis among children keeping histopathology as gold standard. Ann Med Surg. (2018) 38:22–7. 10.1016/j.amsu.2018.11.019 30591836 PMC6305696

